# Health professional regulation in historical context: Canada, the USA and the UK (19th century to present)

**DOI:** 10.1186/s12960-020-00501-y

**Published:** 2020-10-20

**Authors:** Tracey L. Adams

**Affiliations:** Department of Sociology, The University of Western Ontario, London, Ontario N6A 5C2 Canada

**Keywords:** Healthcare professional regulation, History, Canada, US, UK

## Abstract

**Background:**

There is no widespread agreement over what form healthcare professional regulation should take, and the evidence base concerning the effectiveness and fairness of regulatory systems and practices is limited. Those urging policy change argue there is a need to modernize; however, there is much we can learn from reviewing the history of healthcare professional regulation.

**Main body:**

An overview of the history of regulation in Canada, with consideration of the United States of America and the United Kingdom, is provided. Self-regulating professions emerged in the nineteenth century, influenced by a variety of stakeholders responding to local concerns for healthcare quality, access and professional training. Regulatory practices changed over the course of the twentieth and twenty-first centuries in response to changing stakeholders and shifting interests.

**Conclusions:**

Reviewing the history of healthcare professional regulation reveals lessons to inform policy in a range of settings.

## Background

Regulation is necessary to ensure that healthcare professionals provide valued expert services safely and in a manner that benefits the public. Although professional regulation can take many different forms, in the West, it has historically involved legislative mechanisms to govern entry to practice and the conduct of practice of professional occupations. However, there is no widespread agreement over what form healthcare professional regulation should take, and the evidence base concerning the effectiveness and fairness of regulatory practices is limited. Concern has been raised about the effectiveness of prevailing regulatory practices; there are calls for innovation and renewal [1, 2]. The COVID-19 pandemic has further laid bare deficiencies in existing systems [3]. It has become commonplace in some Western countries to argue that “modernization” is needed, as if regulatory solutions can only be found by jettisoning previous practice and starting from scratch. However, there are lessons to learn from the past. Understanding the factors and forces shaping healthcare professional regulation historically can enhance understanding of our present and inform our decisions about the future of regulation.

This paper builds on research on the history of professional self-regulation in Canada, with consideration of the United States of America and the United Kingdom as well [4, 5]. This research explored the regulation of healthcare professions from the mid-nineteenth century to the present, considering how regulation has varied across time and locale and exploring the major concerns, debates and stakeholders shaping regulatory outcomes. Drawing on this research, this paper addresses two questions. First, what were the key considerations and who were the important actors shaping healthcare regulation in the past, and how has that changed? Second, what can we learn from this history to inform discussions of healthcare regulation in the present?

## Main text

### Nineteenth-century healthcare professional regulation

In the United Kingdom, the United States of America and Canada, modern healthcare regulation emerged in the nineteenth century. All three countries fall into what sociologists label the “Anglo-American model” in which organized professionals are empowered by the state to govern their own affairs, especially entry to practice and the conduct of professional practice. According to this account of profession formation, healthcare professionals like medical doctors joined together and used their influence to acquire status, and win legislation granting them the power to self-regulate [6]. Elsewhere, such as in Western Europe, professional regulation was more state-directed [7]. Traditional accounts of professional regulation have tended to over-estimate professional power and underestimate the role of the state and other stakeholders in professional regulation [4, 8]. Nonetheless, professional self-regulation became predominant in Anglo-American countries in the late nineteenth and early twentieth centuries.

Canada, the United States of America and the United Kingdom all had slightly different traditions of healthcare professional regulation. The medical profession was among the first professions to be regulated through statutory legislation and was the most influential, historically. In the United Kingdom, there were a variety of organizations and “colleges” regulating member-practitioners, but no over-arching system until the creation of the General Medical Council in 1858 [6]. The latter was a legislative achievement, following decades of negotiations and effort, and resulting from collaboration among several medical organizations and government leaders, with the goal of establishing some uniformity in medical regulation [8]. In the United States of America, professional regulation actually decreased in the early-to-mid-nineteenth century before medical regulation emerged at the state level in the decades after 1870.

In Canada, medical boards were initially created by state and medical leaders (groups whose membership overlapped). These boards had the power to determine entry to practice, examine candidates and assess their “loyalty, integrity and good morals” [9:22]. However, in Ontario and Quebec (healthcare is a provincial matter in Canada), dissatisfaction with the government spurred regulatory change. In Quebec, medical doctors sought self-regulation in response to unfair treatment and patronage among the British elite that denied opportunity and voice to the French Canadian majority in the province. Here, the fight for fairness and more representative government became intertwined with the drive for professional self-regulation [4]. The latter was achieved in Quebec in the late 1840s, through legislation establishing a College of Physicians and Surgeons of Lower Canada. This body, consisting of all licensed medical practitioners, was granted fairly extensive powers (see Table 1). Ontario passed legislation to create a self-regulating college in 1839. Members of the previous, appointed board of examiners, feeling hard done by the prevailing government, and immigrant medical doctors from Scotland and Ireland, eager for recognition and fairer entry requirements, joined together to seek legislation granting them a greater voice in governing their own affairs. The legislation gave the resulting regulatory college several powers—perhaps too many as they incurred the displeasure of the Royal College of Surgeons in London who appealed to Queen Victoria to disallow the legislation. She did so in 1840 [4]. Not until 1869 was another self-regulating college established; the College of Physicians and Surgeons of Ontario persists today. This college was not profession-created, but was established through legislation by the Ontario government, which devolved considerable powers on the body and made unlicensed practice illegal.

**Table 1:** Medical regulation in the nineteenth century

	United Kingdom	Canada		USA	
		Quebec	Ontario	Illinois	New Mexico
**Date of act**	1858	1847	1839, 1869	1877	1882
**Regulatory body**	General Council of Medical Education and Registration of the United Kingdom	College of Physicians and Surgeons of Lower Canada	College of Physicians of Surgeons of [Upper Canada/Ontario]	State Board of Health	Board of Examiners
**What does the regulatory body do?**	Maintain a register and establish rules for regulating the register. Collect information from other medical bodies and colleges respecting qualifications. Report to the Privy Council.	Power to regulate the study of medicine, surgery, midwifery and pharmacy, and examine candidate credentials, administer oaths, determine qualifications, assess credentials, determine qualifications for entry to practice, determine entry to practice, and establish fees	Maintain and publish a register; review qualifications; examine candidates for entry to practice; regulate self, board of examiners, admission to study or matriculation; and establish a medical school curriculum.	With respect to medical regulation, the board reviews qualifications, examines candidates for registration and hears testimony.	Review diplomas, conduct exams for those without diplomas and review evidence of candidate/practitioner conduct
**Who sits on board?**	Members are appointed by the privy council and various colleges, schools and organizations.	Medical doctors elected by members of the college (CPSLC)	Members elected by members of the college and representatives of the medical schools (1869)	Seven individuals appointed by the governor with the advice of the senate.	Medical doctors (allopaths, homeopaths and eclectic) appointed by the governor
**Is medical practice closed (so only the licensed or registered can practise)?**	No, but the licensed do obtain certain privileges.	Yes	Yes	Yes. Exemption for those in the armed forces	Yes
**Entry to study**	Determined by other parties (colleges and schools)	Knowledge of Latin, history, geography, mathematics and philosophy. Later knowledge of French and English	English language (grammar and composition, arithmetic, algebra, geometry, Latin and one of Greek, French, German or natural philosophy	Not regulated by board	Not regulated by board
**Entry to practice**	Qualifications for practice are determined by pre-existing bodies (like colleges)	Medical diploma, or four years of apprenticeship combined with schooling and exam. Education must be obtained in British dominions. Also evidence of good moral character.	1839—college may set criteria for entry to practice. 1869—previously licensed, medical diploma or pass exam.	Diploma in medicine or completion of board exam	Diploma or exam in specified subjects. Proof of conduct may be reviewed.

Overall, medical regulation in the nineteenth-century Quebec and Ontario, the United Kingdom and many American states was not dissimilar, but meaningful differences are evident (see Table 1).

Variations across region owe much to different social-historical contexts and politics. The provinces of Ontario and Quebec had broader powers, on average, than their UK and American state counterparts. These powers partly reflect different perceptions of professionals in the nineteenth century. American culture tended to view educated elites with suspicion, and Americans were reluctant to grant privileges to some, but not others. In contrast, in Canada, professionals were seen as integral to life in the new colonies and a source of leadership at local, provincial and federal levels [9]. Moreover, there were few schools to educate professionals in Canada, so regulatory bodies there were granted privileges that were largely delegated to existing institutions in the United States of America and the United Kingdom. While the United Kingdom arrived at a legislative solution that built on and worked around a complex organizational landscape, the sweeping powers given to Canadian professions reflect a lack of alternative institutions.

The level at which healthcare professional regulation occurred is also important. In the United States of America and Canada, medical regulation occurred on a regional level and responded to local healthcare needs, concerns, cultures and traditions and local organizations. Even at this level there were concerns that regulatory legislation could be too sweeping. Legislation that restricted the number of practitioners might work fine for urban centres, but leave more remote areas without care providers at all [4]. Regional differences were evidence in the United Kingdom as well, but UK legislation met this challenge by refusing to close the practice of medicine and establishing a model for the GMC that built upon existing institutions and traditions.

When legislators passed legislation granting powers of self-regulation, they articulated several rationales for doing so. In Canada, the emphasis was placed on both professionals’ interests and the public interest. It was deemed to be in the public’s interest to grant authority and privileges to educated, white men who could take on positions of social and community leadership—as long as they were properly educated and trustworthy. Thus, in Canada, by “public interest”, state actors (and professionals) were primarily concerned with ensuring that services were provided by trained and educated people to minimize harm to patients and clients, ensuring sufficient access to services (even if that meant relaxing restrictions on practice by the unlicensed in certain regions), and ethical conduct in practice. In the United States of America, the purpose behind regulation varied somewhat across states, but medical regulation was intended to help consumers distinguish the trained from the untrained, and to enhance public health initiatives [4, 6]. In the United Kingdom, among the rationales provided for regulation were improving service quality, enhancing uniformity and clarifying for the public (and for policy purposes) who was qualified to practice [8].

In all settings, regulatory outcomes were a product of the interplay of interests among legislators, other state actors and professionals. These relationships were not always harmonious. Professional self-regulation at times was prompted by frustration with prevailing governments. Certainly, state actors were often ambivalent and opposed to granting professionals power. They criticized professionals for requesting privileges and questioned how they would use them [4]. For their part, professionals wanted the right to determine their own affairs, and a stronger voice in public policy relating to health [4, 10]. Although they did not always see eye-to-eye, professional self-regulation resulted from the collaboration between the two groups. Professionals’ roles in public life and as advisers on health policy gradually grew. The fact that there was considerable overlap between these groups (some state actors were also professionals) certainly fostered collaboration [4].

Although the system was by no means perfect, it appears that states, citizens and professionals all benefitted to some extent during this era. Less privileged practitioners—the self-trained and untrained—were the main losers from healthcare regulation, especially where practice was closed.

### Twentieth-century changes

The medical profession was not the only self-regulating health profession in the nineteenth century. Pharmacy and, in the United States of America and Canada, dentistry were regulated as well. However, healthcare professional regulation expanded substantially in the twentieth century. The sweeping changes shaping healthcare professional regulation in this era are too many to describe in detail here, but the following developments were crucial: the expansion of health care practice and the regulation of new groups in the early twentieth century; the major influence of the two world wars; the emergence of the welfare state and especially Medicare; concerns over civil rights and justice; rationalization trends including concerns for organizational efficiency; privatization; and interprofessional conflict. Changing relations between professions and the state and the emergence of new stakeholders in the regulatory process strongly shaped regulatory change. Moreover, notions of “the public interest” altered over this period [11].

Exactly who was regulated and how continued to vary cross-nationally, and regionally in Canada and the United States of America, in line with local cultures, regional healthcare movements, organizational developments and state policies. Nursing, physiotherapy and chiropody were among those regulated in all three countries. However, professional groups practising osteopathy, chiropractic and naturopathy (and several other health specialties) emerged more unevenly across the United States of America and Canada. When these healthcare professions were regulated, they were typically granted fewer privileges and responsibilities and had more delimited scopes of practice, and some were not fully self-regulating, but governed by state bodies or boards which could include practitioners from other regulated professions [4, 12].

In many instances, regulation of new healthcare professions aimed to control more than empower. Some of these practices were deemed risky, while for others the main concern was the potential overlap with more established professions. For example, some medical doctors claimed that alternative health practitioners were merely illegal medical practitioners [4, 12]. The fact that these professions were practised by people who were far from elite—working class people in chiropractic, women in nursing and shop-owners in optometry—contributed to legislators’ wariness about delegating regulatory privileges to them.

Whereas in the nineteenth century, regulatory decisions were shaped by negotiations between professionals and legislators, the number of stakeholders shaping regulatory decisions expanded in the early twentieth century. Customers and clients played a significant role—especially in the regulation of alternative healthcare practices like chiropractic, osteopathy and naturopathy and to some extent optometry. In Canada, most state actors were not supportive of chiropractic regulation, but chiropractors’ public support made regulation a politically savvy decision. Consumer concerns that the regulation of optometry would lead to higher-cost goods, in contrast, discouraged optometry regulation and encouraged a form of regulation that was quite controlling. Moreover, healthcare occupations and professions influenced each other’s regulatory outcomes [4, 12]. Medical demands to have a voice in nursing regulation delayed legislation and shaped its form in several regions. Other stakeholders including employers and small business owners also shaped regulatory outcomes. In the early twentieth century, then, professional regulation was the product of negotiations between multiple stakeholders: state actors, professionals seeking regulation or regulatory change, other professionals/practitioners, patients and clients, the judiciary, business interests and employers. As a consequence, regulatory privileges decreased.

In the latter half of the twentieth century, these trends intensified with an increasing number of stakeholders weighing in on each governmental decision. State actors were even more hesitant to expand regulation to new groups, but new groups seeking regulation kept emerging. The implementation of Medicare raised the stakes of healthcare regulation considerably in the United Kingdom (beginning in the 1940s) and Canada (in the 1960s) and spurred rationalization, planning and standardization in professional regulation. In the 1960s and 1970s, the expansion of civil rights movements also impacted professions. In Canada, a federal commission in this area singled out professions as entities reproducing social inequalities and contributing to injustices, especially with respect to access to services as well as access to practice [13]. Upset at the excesses and bad practices of regulators emerged elsewhere too, from the United States of America and United Kingdom to India [6, 14]. Such concerns spurred regulatory change in at least two directions to curb professional power. First, regulatory bodies—like those in the United States of America and Canada—added public members and state actors to regulatory boards, to increase public involvement and decrease abuses of professional power [5]. Second, attempts to standardize regulation and to subject professional bodies to additional oversight increased. In this era, social understandings of the public interest shifted to encompass cost containment and efficiency, along with access and service quality [11]. Employer and other private sector forms of regulation emerged [5]. International regulatory bodies and other organizations like the World Health Organization became more influential as well [15]. Effort was made to make professions and their regulatory bodies more accountable to the publics (and states) they served.

Despite this movement towards standardization, regional variations persisted—and in fact became more prominent in the United States of America and Canada. This is especially seen in the regulation of alternative health professions, like naturopathy, in some regions but not others. Who was regulated and how remained responsive to local health care needs, population size and distribution and other institutional developments [4]. A one-size-fits-all model, where the same professions are regulated in the same way across the country, was not embraced, although educational accreditation was standardized.

In this era, structures of regulation also came to differ. For instance, in the early 1970s, Quebec established a system of co-regulation where professional regulatory bodies are overseen by an overarching body and the Quebec provincial state. In Ontario and some other provinces, healthcare professional regulation focuses on the task—“restricted acts”—and who can perform these tasks. In other regions, healthcare regulation continues to focus on the professional group.

Twentieth century change expanded the number of healthcare professions regulated, and restricted their autonomy—especially in Canada and the United States of America—but problems with self-regulation remained, leading to twenty-first century changes.

### Twenty-first century changes and challenges

In the twenty-first century, healthcare regulation has been completely redrawn in the United Kingdom, Australia and India, to name a few [6, 16]. Oversight of regulatory bodies has increased, while the number of such bodies has decreased. Driving the changes in the United Kingdom and Australia are core principles including health systems sustainability, accountability, transparency, standardization, centralization and a drive for efficiency. These need not be the only principles driving regulatory innovation, however: In India, equity, access and service quality are instead highlighted. Health is a leading economic sector, often constituting over 10% of GDP. Thus, states are eager to ensure this sector is running as efficiently and effectively as possible. Previous forms of professional self-regulation were deemed inefficient and prone to abuses. New systems aim to enhance efficiency, effectiveness and innovation while reducing professional power and autonomy which are regarded as bad for society [17].

In Canada and the United States of America, traditional professional self-regulation has been altered through multiple layers of regulation and oversight (government bodies, professional bodies, employers and so on), but self-regulation has not yet been eliminated [5]. Concerns about regulatory bodies’ transparency, accountability and their disciplinary proceedings—which vary somewhat from one region to the next—are growing, however [1, 6]. There are pressures to increase the scale of regulation too. For instance, in Canada, we are witnessing the amalgamation of regulatory bodies. Additional change is imminent in several Canadian provinces.

The regional nature of professional regulation in Canada and the United States of America—which was arguably beneficial historically providing regulation responsive to local needs and conditions—is increasingly seen as a detriment. It is fragmentary and creates barriers to the flow of healthcare professionals from one region to another. Barriers remain for the internationally qualified as well. Increasing regulation requires international co-operation. In Canada, such concerns intensified during the COVID-19 spring, with a focus on medical and nursing care especially—professions for which national regulation may be possible due to commonalities in provincial entry and education standards [3].

Some of the complaints against professions reflect shifting power and alliances among stakeholders. Professionals, consumers and patients, other occupational practitioners, state actors and private sector interests continue to shape regulatory outcomes, but state actors are increasingly hostile to professions, and seeking to expand employers’ authority at professionals’ expense [17]. In the past, professionals, professional bodies and state actors often worked collaboratively to shape policy and meet healthcare demands and concerns. Members of the two groups shared goals, including the provision of safe, high-quality services to patients and clients and promoting public health. However, this partnership has been breaking down over the last several decades, and profession-government relations are often adversarial [6]. Neo-liberal governance, and the recent rise of populist governments openly skeptical towards expertise, exacerbates hostilities. Professionals are cast as experts seeking self-gain at the expense of others, while the privileges enjoyed by state actors, and the economically and socially advantaged remain unquestioned [17]. Such trends reveal the current crisis of expertise where societies are both reliant upon experts and yet doubt and reject their advice [17].

In this context, conceptualizations of the public interest have shifted once again. The public interest remains a central driving concern of professional regulation, but it is now defined in terms of efficiency, accountability, governance transparency and tight control of professional practice in many countries. In the past, professionals and state actors worked together to raise entry standards to ensure high-quality services, in the public interest. Today, some government agencies define public protection as protecting the public *from* professionals—this rhetoric is most intense in the United Kingdom [1]. In such contexts, professionals are cast as social threats, in need of control. The COVID-19 pandemic may challenge this narrative, but it is too soon to tell.

In this period of change, concerns over healthcare professional regulation continue, but with a limited evidence base on effective regulation, and changing social values, it is not clear what the future will bring.

## Conclusions

Recent trends in professional healthcare regulation reject the past in favour of modernization; however, reviewing the history of self-regulation points to several lessons that could inform policy in a range of settings around the world.

- The aims of regulation are multiple—including health system sustainability, public protection, quality service provision, ethical practice, efficiency and effectiveness, transparency, accountability, flexibility and so on. Balancing these many goals may be challenging, but doing so is vitally important.
- While it is important to seek out best practices, a one-size-fits-all model may not always be appropriate. Healthcare regulation has historically addressed local concerns and developments, and there are times when the flexibility that a local focus can provide is important for enhancing service quality and access, while upholding local cultures and meeting local healthcare needs. International regulation, or importing regulatory structures from elsewhere, can be problematic if it does not meet regional needs and cultures.
- Understandings of the public interest are socially constructed, varying across time and place. Policymakers may benefit from identifying core principles and goals, and designing regulatory measures to meet those goals. Regulatory systems that aim to raise service quality may look different from those prioritizing efficiency.
- Many stakeholders have shaped regulation historically, and regulation may work best when stakeholders work collaboratively. Traditional schemes of professional self-regulation granted some professionals considerable power, but regulatory systems that privilege the interests of government leaders, employers or other powerholders can be equally flawed. Good regulatory policy should consider the voices and concerns of all stakeholders and develop regulatory solutions that work for service providers, service users and others, while providing oversight and accountability. Successful regulation appears to require collaboration.

In conclusion, countries must consider many factors when regulating. Looking at regulatory best practices is beneficial, but since professional regulation balances a variety of goals, local needs, cultures, service providers and politics, regulatory solutions in one context may not translate to, or succeed within, others.

## Data Availability

Data are publically available and key sources are cited.
